# Inhibiting STAT3 signaling is involved in the anti-melanoma effects of a herbal formula comprising Sophorae Flos and Lonicerae Japonicae Flos

**DOI:** 10.1038/s41598-017-03351-2

**Published:** 2017-06-08

**Authors:** Ting Li, Xiuqiong Fu, Anfernee Kai-Wing Tse, Hui Guo, Kin Wah Lee, Bin Liu, Tao Su, Xueyu Wang, Zhiling Yu

**Affiliations:** 1 Center for Cancer and Inflammation Research, School of Chinese Medicine, Hong Kong Baptist University, Kowloon Tong, Hong Kong, China; 2 Research and Development Centre for Natural Health Products, HKBU Shenzhen Research Institute and Continuing Education, Shenzhen, China

## Abstract

A herbal formula (SL) comprising Sophorae Flos and Lonicerae Japonicae Flos was traditionally used to treat melanoma. Constitutively active signal transducer and activator of transcription 3 (STAT3) has been proposed as a therapeutic target in melanoma. Here we investigated whether an ethanolic extract of SL (SLE) exerted anti-melanoma activities by inhibiting STAT3 signaling. B16F10 allograft model, A375 and B16F10 cells were employed to assess the *in vivo* and *in vitro* anti-melanoma activities of SLE. A375 cells stably expressing STAT3C, a constitutively active STAT3 mutant, were used to determine the role of STAT3 signaling in SLE’s anti-melanoma effects. Intragastric administration of SLE (1.2 g/kg) potently inhibited melanoma growth in mice and inhibited STAT3 phosphorylation in the tumors. In cultured cells, SLE dramatically reduced cell viability, induced apoptosis, suppressed migration and invasion, and restrained STAT3 activation and nuclear localization. STAT3C overexpression in A375 cells diminished SLE’s effects on cell viability, apoptosis and invasion. Collectively, SLE exerted potent anti-melanoma effects partially by inhibiting STAT3 signaling. This study provides pharmacological justification for the traditional use of this formula in treating melanoma, and suggests that SLE has the potential to be developed as a modern alternative and/or complimentary agent for melanoma treatment and prevention.

## Introduction

Melanoma, a highly malignant neoplasm of the melanocytes, is the most aggressive form of skin cancer^1^. It accounts for less than 5% of all skin cancer cases, but the vast majority (80%) of skin cancer related-deaths^2^. The incidence of malignant melanoma has been increasing at a steady rate in fair-skinned populations around the world for decades^3, 4^. However, currently available chemotherapeutics against malignant melanoma are often expensive, with toxic side effects, low response rates, and/or high tendency to develop tolerance^5–8^. These disappointing but harsh realities highlight the urgency of exploring novel, safe and effective alternative approaches for melanoma management. Because of their biological activity and low toxicity, natural products (i.e., food, herbs) have been demonstrated to be promising candidates for melanoma prevention and treatment^9, 10^. Signal transducer and activator of the transcription 3 (STAT3), which is constitutively activated at 50 to 90% frequencies in diverse human cancers including melanoma, has been considered as a potential target for melanoma treatment^11^. Previous experimental findings have demonstrated that targeting STAT3 in melanoma tumor models induces tumor cell death/tumor regression^12, 13^ and inhibits metastasis^14^.

Sophorae Flos (SF), the flower and flower-bud of *Sophora japonica* L., is commonly consumed as a vegetable and used to make jam and snacks in China. It has skin-care benefits^15^. The dye extract from SF has been shown to possess ultraviolet protective properties^16^. Lonicerae Japonicae Flos (LJF), the flower bud of *Lonicera japonica* Thunb., is widely consumed as tea, and has long been used for treating skin carbuncles and pyocutaneous diseases in Asian countries^17, 18^. Both SF and LJF are commonly used in treating melanoma in traditional Chinese medicine (TCM) practice^19–21^. In *Yi Xue Qi Meng* (a Chinese medicine classic issued 600 years ago), a formula (SL) consisting of SF and LJF simmered in rice wine is documented as a remedy for subcutaneous ulcer, skin carbuncle and abscess, which have TCM symptoms resembling those of melanoma. In addition, constituents in SF and LJF, such as rutin, quercetin and luteolin, have been shown to possess anti-melanoma properties^22–24^. Some of these constituents have also been demonstrated to inhibit STAT3 signaling in different types of tumor cells^23, 25^. However, there is no report about the pharmacological effect of SL on melanoma so far. In the present study, we evaluated the *in vivo* and *in vitro* anti-melanoma effects of an ethanolic extract of SL (SLE). Human A375 and murine B16F10 melanoma cells, together with the B16F10 melanoma allograft model in C57/BL6 mice were employed. The involvement of STAT3 signaling in the anti-melanoma effects of SLE was also explored.

## Results

### SLE restrained tumor growth and STAT3 activation in a B16F10 allograft model

**Figure 1:**
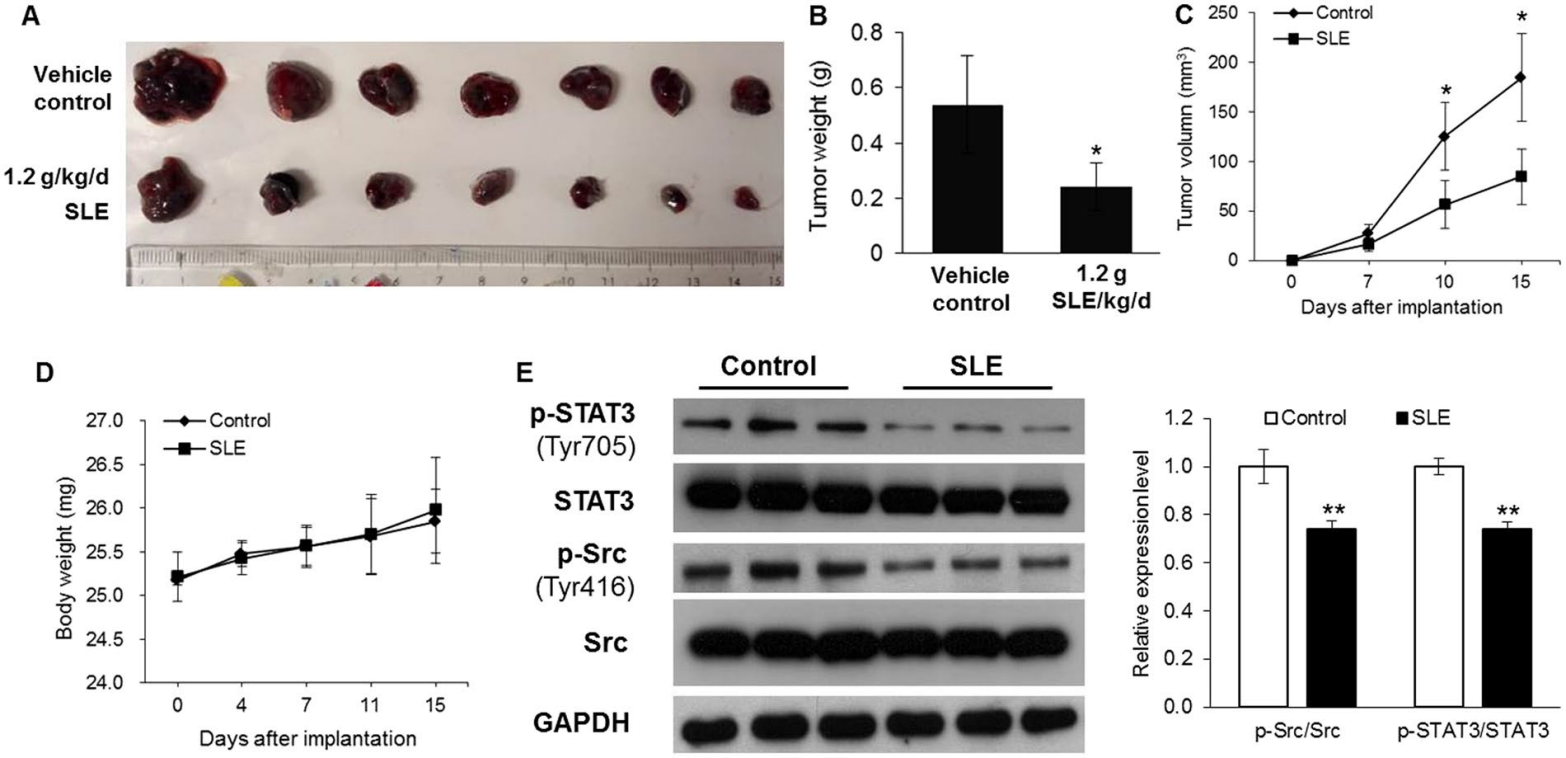
Anti-melanoma effects of SLE in mice. (**A**) The photo of B16F10 tumors dissected from mice. (**B**) Mean weights of the dissected tumors. (**C**) Time-dependent effect of SLE on melanoma growth in a B16F10 allograft mouse model. (**D**) Body weights at different time points. In (**B**), (**C**) and (**D**), data were means ± SD of 7 mice. (**E**) Protein expression levels of phospho-STAT3 (Tyr705), STAT3, phospho-Src (Tyr416), Src and GAPDH in B16F10 tumors collected from 3 individual mice were examined by immunoblotting (left panel) and the relative band intensity was analyzed by Image J software (right panel). The images shown were the cropped blots for the corresponding proteins. The original blots from which images were cropped were shown in Supplementary Dataset. *P < 0.05, **P < 0.01 versus vehicle control group.

The *in vivo* anti-melanoma effect of SLE was evaluated using a B16F10 melanoma allograft C57/BL6 mouse model. At the end of the experimental period, each mouse only had one tumor. As shown in Fig. 1A, daily intragastric administration of 1.2 g/kg SLE for 15 days significantly inhibited tumor growth in mice. In comparison with the control group, the average tumor size and tumor weight in SLE-treated group were remarkably reduced by 54.1% and 55.3% after SLE intervention, respectively (Fig. 1B and C). No animal death occured during the experimental period. No abnormalities were found in all mice at necropsy on day 15. No significant differences were observed in the food and water consumption (Data not shown), and body weight (Fig. 1D) between the two groups. It is well recognized that constitutive activation of STAT3 plays a critical role in melanoma development^10^. To determine whether SLE affects STAT3 activation, we examined the expression of phosphorylated STAT3 in tumor tissues by immunoblotting. SLE potently decreased the protein levels of phospho-STAT3 (Tyr705). SLE also inhibited the expression of phospho-Src (Tyr416) in tumor tissues. The non-receptor tyrosine kinase Src is an upstream kinase of STAT3. SLE did not affect the expressions of total STAT3 and total Src in B16F10 allograft tumors (Fig. 1E).

### SLE exhibited higher cytotoxicity in melanoma cells than in normal skin cells

**Figure 2:**
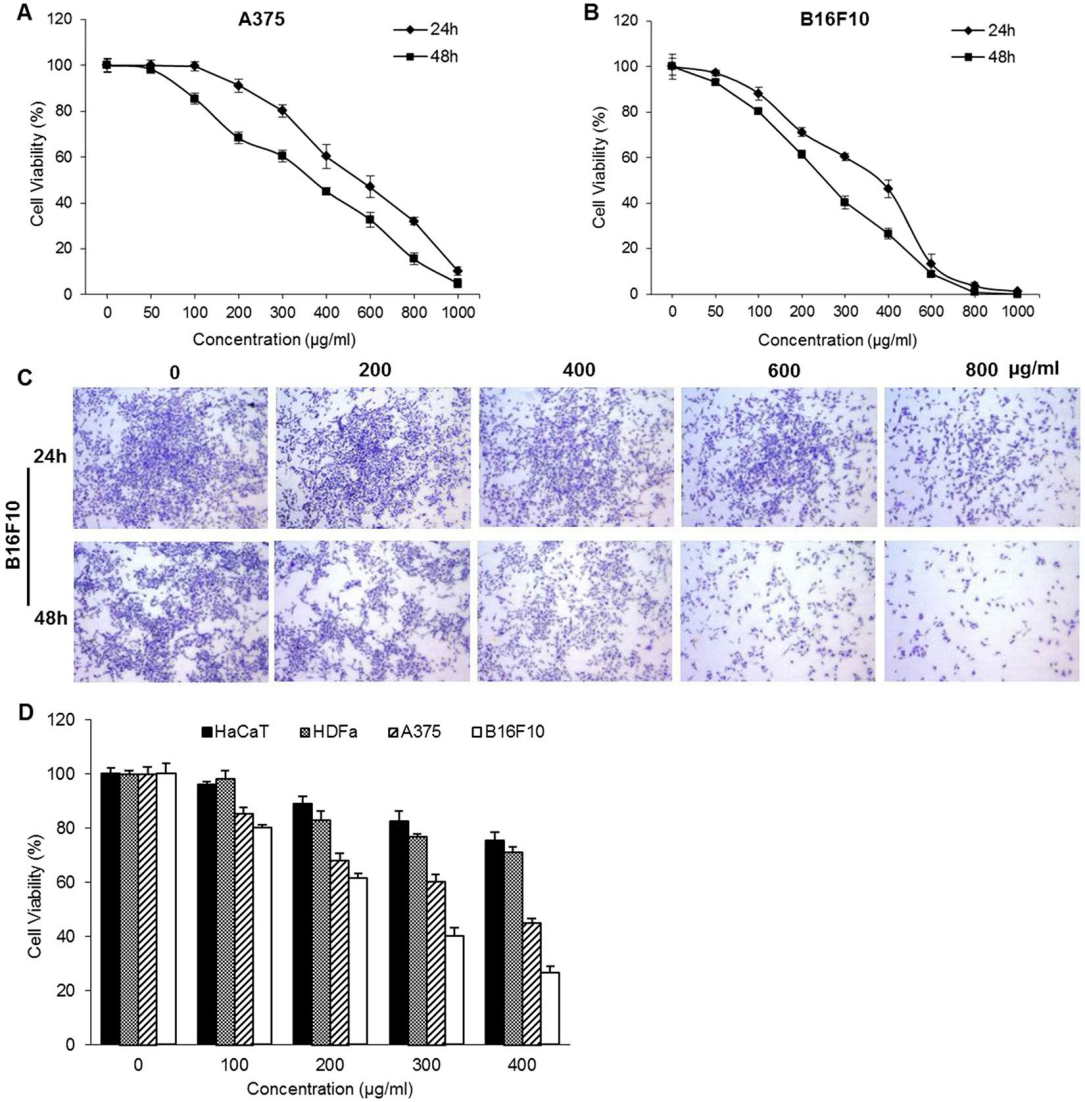
Cytotoxic effects of SLE on melanoma cells and normal skin cells. Effects of SLE on the viabilities of A375 cells (**A**) and B16F10 cells (**B**) were determined by the MTT assay and trypan blue exclusion assay, respectively. (**C**) Proliferation inhibitory effects of SLE on B16F10 cells measured by the crystal violet staining assay. Cells were treated with the indicated concentrations of SLE for 24 or 48 h. (**D**) Comparison of viability inhibitory effects of SLE on melanoma cells and normal skin cells. Cells were treated with the indicated concentrations of SLE for 48 h. Data were presented as means ± SD of three independent experiments.

SLE treatment (50, 100, 200, 300, 400, 600, 800 and 1000 μg/mL, 24 and 48 h) decreased the viabilities of A375 and B16F10 cells in time- and dose- dependent manners (Fig. 2A and B). The IC_50_ values of SLE were 321.60 ± 3.45 μg/mL against A375 cells and 281.29 ± 5.15 μg/mL against B16F10 cells at 48 h. The crystal violet staining assay visually validated the inhibitory effects of SLE on B16F10 cell proliferation (Fig. 2C). Moreover, the inhibition rates of 100, 200, 300 and 400 μg/mL of SLE on human HDFa and HaCaT normal skin cells were much lower than that on A375 and B16F10 melanoma cells (Fig. 2D).

### SLE induced apoptosis of A375 human melanoma cells

**Figure 3:**
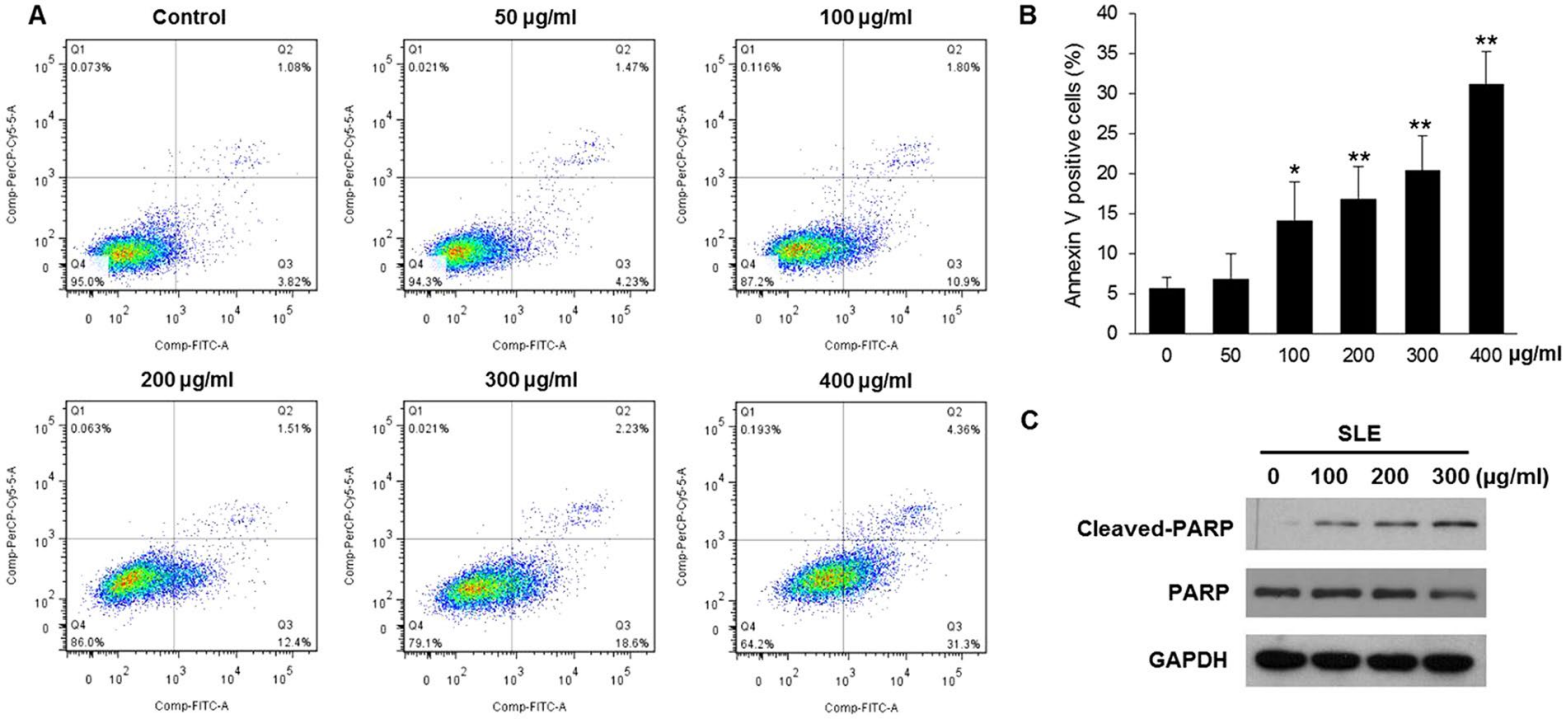
Pro-apoptotic effects of SLE on A375 melanoma cells. Cells were treated with the indicated concentrations of SLE for 48 h. (**A**) Representative flow cytometry plots of cell apoptosis. Apoptosis was analyzed using the Annexin V/7-AAD double staining assay. (**B**) The percentage of apoptotic cells after SLE treatment. Data were shown as means ± SD of three independent experiments. *P < 0.05, **P < 0.01 versus control. (**C**) Effects of SLE on PARP cleavage in A375 cells. Protein levels were examined by immunoblotting and the cropped blots were shown. GAPDH was included as a loading control. Original uncropped western blot images were shown in Supplementary Dataset.

Annexin V/7-AAD double staining assay was performed to quantify the apoptotic effects of SLE on A375 cells. SLE treatment for 48 h dose-dependently induced apoptosis in A375 cells. SLE at the doses of 100, 200, 300 and 400 μg/mL significantly increased the rate of Annexin V positive cells to 14.2 ± 4.7%, 16.9 ± 4.0%, 20.4 ± 4.3% and 31.2 ± 4.0%, respectively, as compared with the untreated cells (5.7 ± 1.3%) (Fig. 3A and B). To further validate the pro-apoptotic effects of SLE, we determined the impact of SLE on the cleavage of poly (ADP-ribose) polymerase (PARP), which is considered as a hallmark of apoptosis. SLE (100, 200 and 300 μg/mL) dose-dependently increased the protein levels of cleaved-PARP in A375 cells (Fig. 3C).

### SLE suppressed the migratory and invasive abilities of melanoma cells

**Figure 4:**
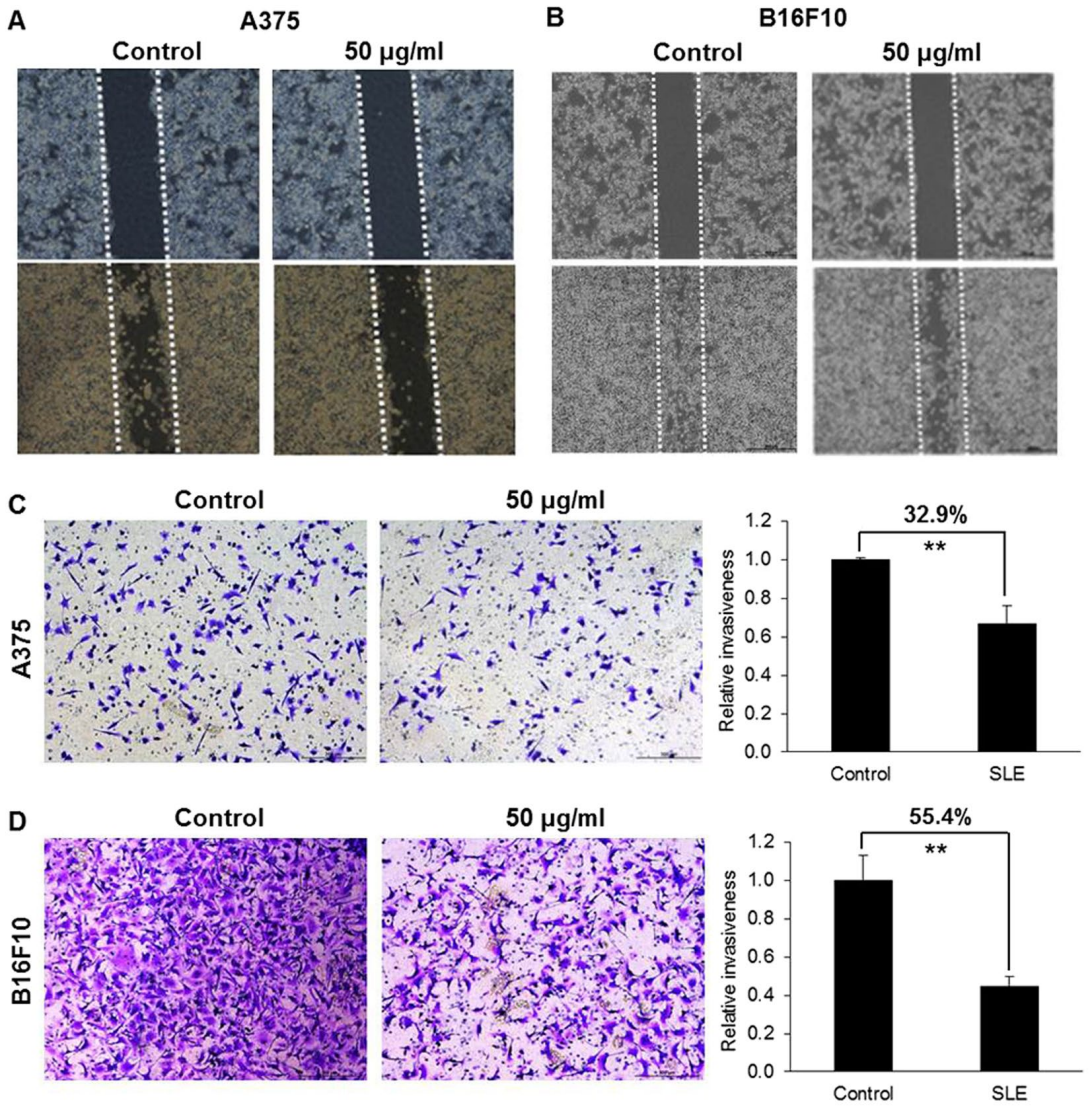
Inhibitory effects of SLE on melanoma cell migration and invasion. Representative photographs of A375 (**A**) and B16F10 (**B**) cell migration after SLE (50 μg/mL) treatment. Cells seeded in 6-well plates were grown to 80–90% confluence. A single scratch was made in the cell monolayer, and followed by vehicle control or SLE treatment for 24 h. Pictures were taken at 0 and 24 h after treatment. Effects of SLE on A375 (**C**) and B16F10 (**D**) cell invasion were examined using the matrigel invasion assay. Representative photographs of invaded cells (left) and quantification of invasiveness (right) were shown. Cells (1.5 × 10^5^) in DMEM-0.1% BSA with SLE (50 μg/mL) or vehicle control were allowed to pass through matrigel coated membrane for 24 h. Cells on the lower surface of the membrane were considered as invaded cells. Number of invading cells was determined as the average cell number in 5 random microscope areas for each condition for each separate replicate experiment. Data were shown as mean ± SD from three independent experiments, **P < 0.01.

Wound healing assay and matrigel invasion assay were performed to determine the effects of SLE on melanoma cell migration and invasion, respectively. As shown in Fig. 4A and B, SLE (50 μg/mL) treatment for 24 h significantly inhibited the migration of A375 and B16F10 cells. For the cell invasion assay, A375 and B16F10 cells with or without SLE treatment were allowed to invade for 24 h. 50 μg/mL of SLE markedly reduced the cell invasiveness by 32.9% for A375 cells and 55.4% for B16F10 cells (Fig. 4C and D). As shown in cell viability assays, SLE at 50 μg/mL did not significantly affect the viabilities of A375 and B16F10 cells (Fig. 2A and B).

### SLE inhibited STAT3 activation and STAT3 nuclear localization in melanoma cells

**Figure 5:**
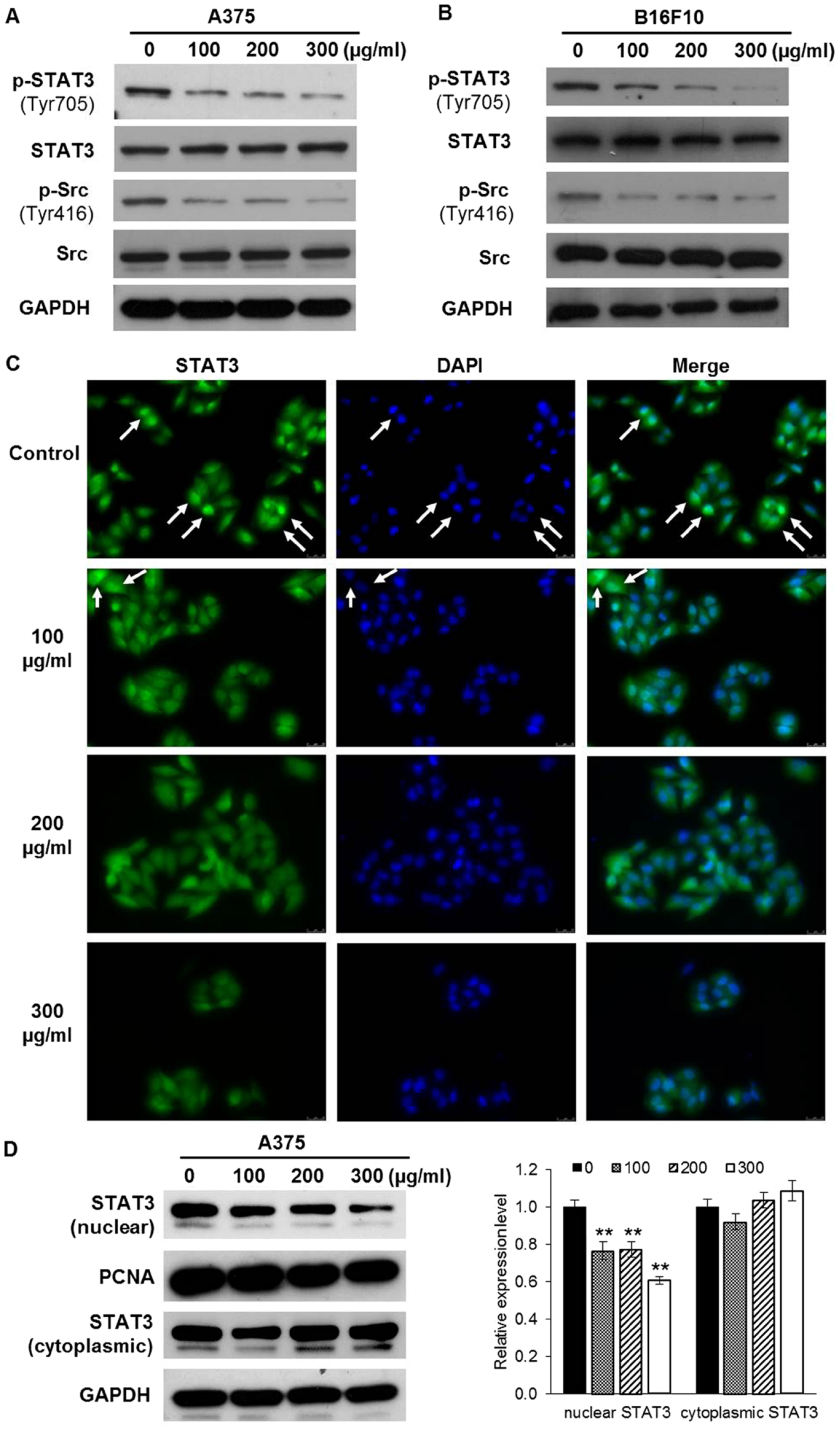
SLE suppressed STAT3 and Src phosphorylation, and STAT3 nuclear localization in melanoma cells. (**A**) The expression levels of phospho-STAT3 (Tyr705), STAT3, phospho-Src (Tyr416) and Src in A375 (**A**) and B16F10 (**B**) cells. Cells were treated with the indicated concentrations of SLE for 48 h. Protein levels were examined by immunoblotting. GAPDH was used as a loading control. (**C**) Immunofluorescence staining of STAT3 in A375 cells treated with SLE for 48 h. Cells were fixed and labeled with STAT3 antibody and counterstained with DAPI. (**D**) Immunoblot analysis of STAT3 levels in cytoplasmic and nuclear fractions of A375 cells treated with SLE for 48 h (left panel). GAPDH represents a loading control for the cytoplasmic fraction, and PCNA represents a loading control for the nuclear fraction. Relative protein levels were analyzed by Image J software (right panel). Results were shown as mean ± SD from three independent experiments, **P < 0.01. The corresponding western blot images represented were cropped sections and the original images were shown in Supplementary Dataset.

Immunoblotting assay was employed to further determine the effects of SLE on STAT3 activation in melanoma cells. As expected, SLE (100, 200 and 300 μg/mL) treatment for 48 h apparently suppressed the phosphorylation of STAT3 (Tyr705) and Src (Tyr 416), but did not affect total STAT3 and total Src expressions, in both A375 and B16F10 cells (Fig. 5A and B). After phosphorylation, STAT3 homodimerizes and translocates into the nucleus where it binds to promoter elements of responsive target genes to regulate their transcriptions^26^. Hence, we examined whether SLE could affect STAT3 nuclear localization. The immunofluorescence images and immunoblotting results showed that SLE at 100, 200 and 300 μg/mL caused evident decreases in nuclear STAT3 levels in A375 cells (Fig. 5C and D).

### SLE inhibited the expression of STAT3 target genes in melanoma cells

**Figure 6:**
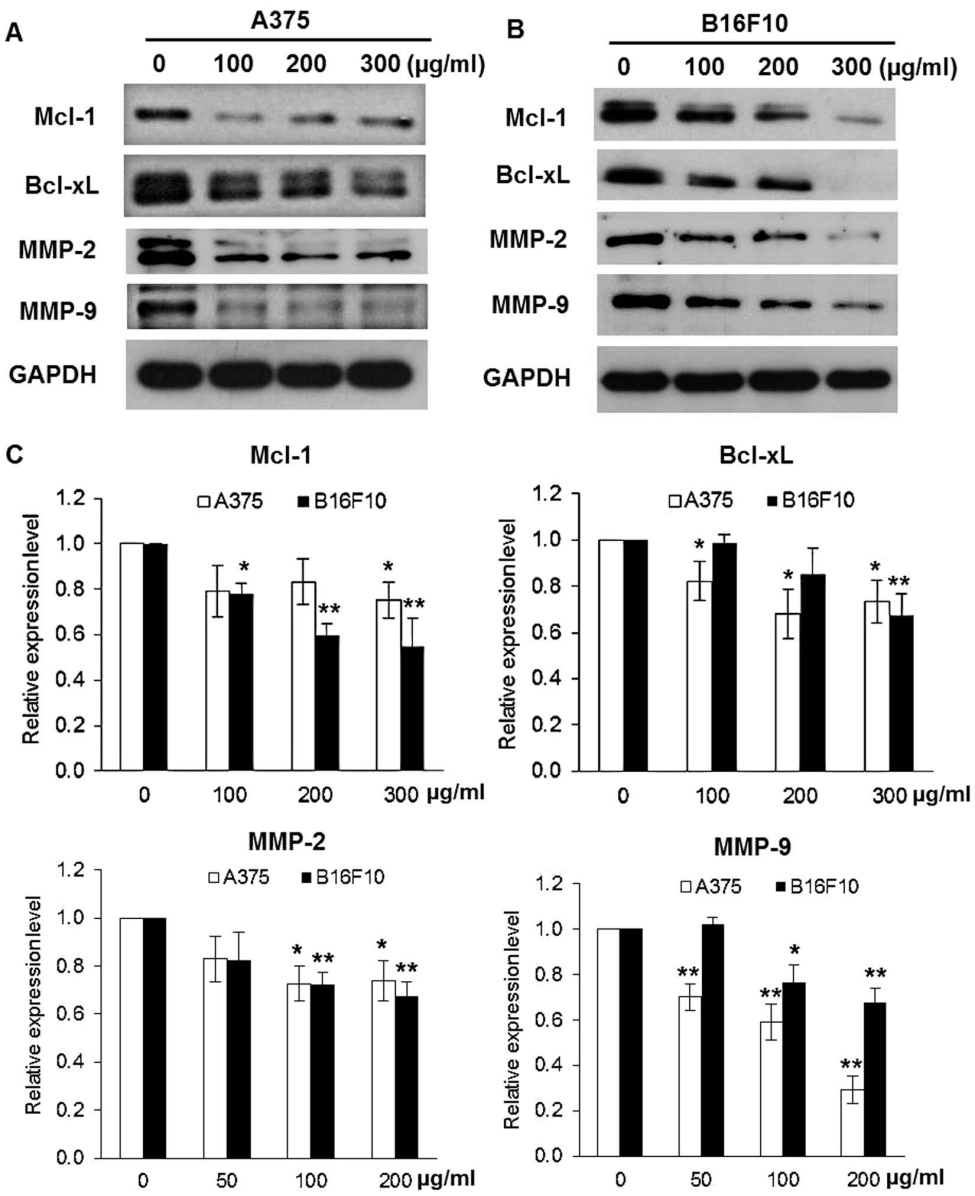
SLE downregulated the expression levels of STAT3 target genes in melanoma cells. Protein expressions of Mcl-1, Bcl-xL, MMP-2 and MMP-9 in A375 (**A**) and B16F10 (**B**) cells. Cells were treated with indicated concentrations of SLE for 48 h. Protein levels were examined by immunoblotting and cropped blots were displayed. The original blot images were shown in Supplementary Dataset. (**C**) The mRNA expressions of Mcl-1, Bcl-xL, MMP-2 and MMP-9 in A375 and B16F10 cells. Cells were treated with indicated concentrations of SLE for 24 h. Total RNA was extracted with Trizol. Gene expression levels were detected using RT-PCR analysis. Data were shown as fold change ± SD of three independent experiments, *P < 0.05, **P < 0.01 versus vehicle control.

Mcl-1 and Bcl-xL are STAT3-target genes involved in cell survival, and matrix metalloproteinase (MMP)-2 and MMP-9 are STAT3-target genes involved in cell migration and invasion^11^. As shown in Fig. 6A and C, SLE effectively decreased the mRNA and protein levels of Mcl-1, Bcl-xL, MMP-2 and MMP-9 in A375 cells. SLE also dose-dependently inhibited mRNA and protein expressions of Mcl-1, Bcl-xL, MMP-2 and MMP-9 in B16F10 cells (Fig. 6B and C). These data further suggest that STAT3 signaling may be involved in the anti-melanoma action of SLE.

### Overexpression of STAT3C in A375 cells diminished the effects of SLE on cell viability, apoptosis and invasion

**Figure 7:**
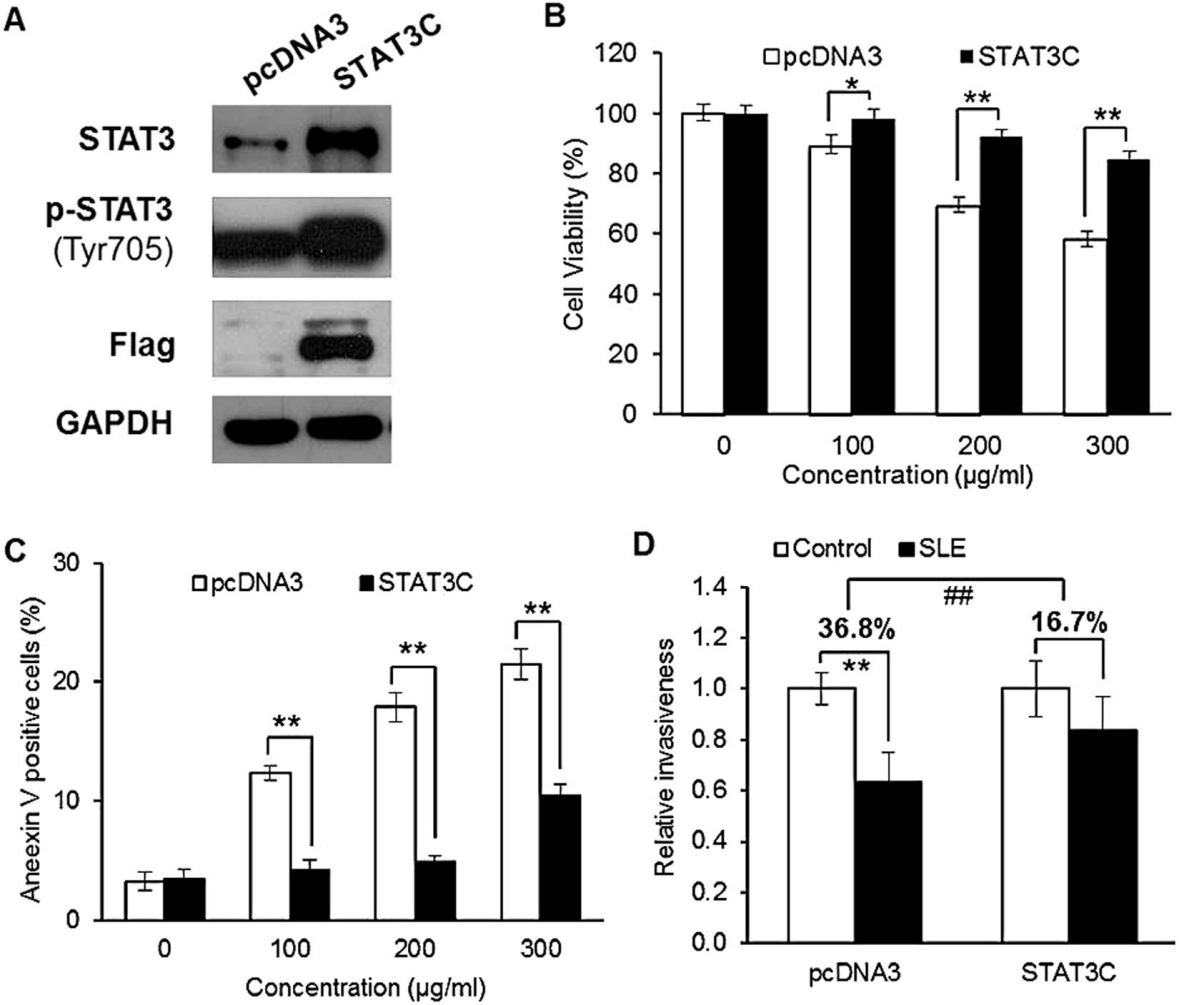
Overexpression of STAT3C in A375 cells diminished the effects of SLE on cell viability, apoptosis and invasion. (**A**) Expression levels of phospho-STAT3 (Tyr705), STAT3 and Flag in A375 cells stably expressing Flag-tagged STAT3C or empty vector (pcDNA3). GAPDH was included as a loading control. Proteins from cultured cells were examined by immunoblotting and cropped blot images were shown. The original uncropped images were shown in Supplementary Dataset. Results shown were the representatives of three independent experiments. (**B**) Effects of STAT3C overexpression on SLE-mediated cytotoxic activity. (**C**) Effects of STAT3C overexpression on SLE-mediated apoptotic effects. In (**B**) and (**C**), A375 cells stably expressing STAT3C or empty vector were treated with indicated concentrations of SLE for 48 h. Cell viability was measured by the MTT assay. Apoptosis was determined by flow cytometric analysis of cells double stained with Annexin V/7-AAD. Data were presented as the mean ± SD of three independent experiments, *P < 0.05, **P < 0.01. (**D**) Effects of STAT3C overexpression on SLE-mediated inhibitory effects on cell invasion. A375 cells stably transfected with STAT3C or vector were treated with SLE (50 μg/mL) or vehicle control for 24 h. Cell invasion was determined by matrigel invasion assay. Statistical analyses were performed by ANOVA followed by LSD Test. **P < 0.01, SLE treatment versus vehicle control treatment. ^##^ *P* < 0.01, SLE-mediated cell invasion inhibition in A375 cells stably transfected with STAT3C versus that in A375 cells stably transfected with empty vector.

To confirm the involvement of STAT3 signaling in SLE’s anti-melanoma effects, A375 cells were stably transfected with a plasmid containing STAT3C, a constitutively active variant of STAT3. Immunoblotting results showed that transfection of STAT3C caused remarkable elevation in both total STAT3 and phospho-STAT3 (Tyr705) levels, compared to transfection with an empty vector (Fig. 7A). The effects of SLE on the viability, apoptosis and invasion of A375 control cells and cells stably expressing STAT3C were compared. When treated with 100, 200 and 300 μg/mL of SLE for 48 h, the viability inhibition rate was significantly decreased from 10.78 ± 3.64%, 30.88 ± 2.86%, 41.54 ± 2.43% in A375 cells transfected with empty vector to 1.38 ± 2.76%, 7.28 ± 2.04%, 15.24 ± 2.75% in cells transfected with STAT3C, respectively (Fig. 7B). Overexpression of STAT3C evidently diminished the apoptotic effects of SLE on A375 cells (Fig. 7C). STAT3C overexpression in A375 cells also remarkably attenuated 50 μg/mL of SLE-induced cell invasion inhibition rate from 36.8% to 16.7% (Fig. 7D).

## Discussion

Although mutant BRAF targeted-therapy and immunotherapy are showing exciting clinical results, the 5-year survival rate for patients with distant metastatic melanoma is merely 17%^4^. Alternative management options for melanoma are urgently needed. TCM-based herbs, especially edible herbs, have been recognized as promising resources for alternative/complementary approaches for cancer management^9, 10^. In a TCM classic *Yi Xue Qi Meng* written 600 years ago, a herbal formula (SL) consisting of SF and LJF was documented to be used by Chinese medicine practitioners to treat skin diseases with TCM symptoms that resemble melanoma. However, there is no modern clinical or experimental evidence about the anti-melanoma action of this formula. In this study, we demonstrated that SLE, an ethanolic extract of SL, exerted potent prophylactic effects on melanoma growth in B16F10 melanoma-bearing mice. It is generally believed that the impacts of food or food products on diseases are modest. However, we observed that the treatment with 1.2 g/kg of SLE for 15 days exerted potent anti-melanoma effects in animals. Specifically, oral administration of SLE caused 54.1% and 55.3% reduction in the average tumor size and tumor weight in mice, respectively. SLE, utilized in this study, was prepared with 30% ethanol. This was based on the original documentation in a TCM classic that the formula SL (SF and LJF in a 5/1 ratio) is decocted with rice wine (about 30% alcohol) for treating skin disorders. The dose of 1.2 g/kg of SLE used for the *in vivo* experiment is the human equivalent dose. Whether SLE treatment with higher dose and longer duration exhibits more potent anti-melanoma effects is a question to be addressed. It is noteworthy that 1.2 g/kg of SLE did not cause observable reduction in animals’ body weight (Fig. 1D) and any other abnormalities in clinical signs and gross pathology, suggesting that SLE has very low toxicity. Furthermore, *in vitro* MTT assays showed that SLE exhibited much lower cytotoxicity on human normal skin cells than on tumorous A375 cells (Fig. 2D). Indeed, SF and LJF are consumed as vegetable and tea, respectively, in Asian countries, indicating their edibility and safety^27^. These suggest that 1.2 g/kg of SLE is effective and safe for melanoma prevention. Further studies are warranted to identify the bioactive constituents responsible for the anti-melanoma activity of SLE. We hope to discover compounds with potent anti-melanoma efficacy from SLE, just like the discovery of artemisinin from a traditional anti-malaria Chinese medicinal herb Herba Artemisiar Annuae, which was inspired from a record in a TCM classic.

In our previous studies, it was found that quercetin, one of the bioflavonoids occurring in both SF and LJF, exhibited anti-melanoma action partially by inhibiting STAT3 signaling^23^. In this study, SLE treatment also effectively inhibited the phosphorylation of STAT3 and one of its upstream kinases Src in B16F10 tumors. STAT3, persistently activated in melanoma, has been shown to regulate the transcription of a panel of genes involved in several oncogenic features, such as cell proliferation, apoptosis and metastasis^28^. Therefore, we evaluated the potential effects of SLE on the viability, apoptosis, migration and invasion of human A375 and murine B16F10 cells. Our results showed that SLE exhibited potent *in vitro* anti-melanoma effects, as evidenced by the significant inhibition of cell viability, migration and invasion, as well as the induction of apoptosis in melanoma cells treated with SLE.

In melanoma cells, we also found that SLE obviously suppressed the phosphorylation of STAT3 (Tyr 705) and Src (Tyr 416), which is consistent with the results of our animal experiments. STAT3 is a cytoplasmic transcription factor that transmits signals from plasma membrane to nucleus^26^. It contains Src homology 2 (SH2) domains and can be transcriptionally activated by Src family kinases through phosphorylating its tyrosine 705 residue^29, 30^. Upon phosphorylation, STAT3 forms homodimers, which translocate into the nucleus to mediate the transcription of STAT3 target genes^26^. Thus, we speculated that SLE-triggered inhibition of STAT3 Tyr 705 phosphorylation should cause reduction of STAT3 nuclear localization and suppression of STAT3 target genes expression. STAT3 contributes to melanoma growth by targeting the antiapoptotic proteins Bcl-xL and Mcl-1, which are upregulated during melanoma progression^11, 31^. STAT3 activation also promotes melanoma metastasis *via* increasing the expressions of vascular endothelial growth factor (VEGF), MMP-2 and MMP-9^14, 32–34^. We found that SLE inhibited nuclear accumulation of STAT3 in A375 cells, decreased the protein and mRNA levels of Mcl-1, Bcl-xL MMP-2 and MMP-9 in both A375 and B16F10 cells; whereas, SLE did not reduce the expression of VEGF (Data not shown). These results demonstrate that the suppressive capabilities of SLE on melanoma cell survival, migration and invasion may be attributed to the inhibition of STAT3-targeted Mcl-1, Bcl-xL, MMP-2 and MMP-9. To further validate the contribution of STAT3 signaling in the anti-melanoma effects of SLE, we established A375 stable cells persistently expressing STAT3C, a constitutively active variant of STAT3. We found that STAT3C overexpression in A375 cells significantly diminished the inhibitory effects of SLE on cell viability and invasion, and also the pro-apoptotic effects of SLE. Furthermore, MTT assays showed that the cytotoxic effect of SLE could be observed in various melanoma cell lines with different genetic backgrounds, and the effect on p-STAT3-positive cells was stronger than on p-STAT3-negative cells (see Supplementary Fig. 1). These results demonstrate that SLE exerts anti-melanoma effects, at least in part, by inhibiting STAT3 signaling. One of the major challenges in developing targeted cancer therapies is reducing or eliminating the severe adverse events resulting from on-target and off-target effects^35, 36^. On-target side effects refer to the pharmacodynamic effect on normal tissues, and off-target side effects are unexpected toxicities derived from the inhibition of unintended or unknown functions^36^. For specific STAT3 inhibitors (WP1066, AZD9150, STAT3 DECOY, OPB-31121 and OPB-51602), several clinical trials have been approved to use them to treat melanoma and other tumors (https://clinicaltrials.gov/). However, some trials have been discontinued due to the severe adverse events^37–39^. The herbal formula SL has long been used in melanoma management by TCM practitioners in ancient China without unfavorable effects reported, and our experimental findings indicate that SLE is effective and safe for fighting melanoma. These are probably the comprehensive effects of SLE with its multicomponent and multitarget natures, which are exactly the advantages of TCM formula. Therefore, we deem that SLE may have greater clinical significance compared to specific STAT3 inhibitors.

Considering the multitarget characteristic of SLE, we believe that STAT3 is one of the targets of SLE. To find more molecular targets of SLE, we analyzed the differentially expressed genes in B16F10 tumors collected from mice with or without SLE treatment using transcriptome re-sequencing analysis. We found that SLE altered the expressions of some genes related to tumor immune evasion (e.g. IL-6, IL-17, STAT1, TNF-α, CD45, MHCII) (Data not shown). In the future, therefore, we will investigate the impact of SLE on melanoma immune microenvironment.

Our results showed that a clinically relevant dose of SLE suppressed melanoma growth in a mouse model. SLE also inhibited cell viability, induced cell apoptosis, restrained cell migration and invasion, suppressed STAT3 activation and nuclear localization, and downregulated STAT3 target genes in melanoma cells. Overexpression of constitutively active STAT3C diminished these effects. Thus, we conclude that SLE, an ethanolic extract of a herbal formula comprising SF and LJF, exerts anti-melanoma effects, and these effects are partially due to the inhibition of STAT3 signaling. This study provides pharmacological justification for the traditional use of this formula in melanoma treatment, and suggests that SLE and SLE-derived compounds have the potential to be developed as modern alternative and/or complimentary agents for melanoma management. Based on the ancient record and our experimental data, we recommend clinical trials of SLE for melanoma prevention and treatment.

## Methods

### Preparation of SLE

SF and LJF were purchased from the Mr. & Mrs. Chan Hon Yin Chinese Medicine Specialty Clinic and Good Clinical Practice Centre in Hong Kong Baptist University. Both of them were authenticated by the corresponding author. Voucher specimens have been deposited at the Centre for Cancer and Inflammation Research, School of Chinese Medicine, Hong Kong Baptist University. It is documented in *Yi Xue Qi Meng* that SF and LJF (at a ratio of 5:1) are mixed and decocted with rice wine (about 30% alcohol) for treating skin disorders, suggesting that some active constituents are lipophilic. Therefore, we extracted the mixture of SF and LJF (at a 5/1 ratio) with 30% (v/v) ethanol aqueous by heat refluxing. The obtained ethanolic extract of SL (SLE) was then lyophilized with a Virtis Freeze Dryer (The Virtis Company, New York, USA). The yield of powdered SLE was 8.49%. The contents of rutin and chlorogenic acid (see Supplementary Fig. 2) in SLE was quantified by HPLC analyses.

### Animal experimentation

Male C57/BL6 mice (6 weeks old) were obtained from The Laboratory Animal Services Centre, The Chinese University of Hong Kong. All care and handling of animals were performed with the approval of the Department of Health, Hong Kong. Experimental procedures were approved by the Committee on the Use of Human & Animal Subjects of the Hong Kong Baptist University. B16F10 cells (1 × 10^6^) were resuspended in 0.1 mL of phosphate-buffered saline (PBS) and inoculated subcutaneously into the backs of C57/BL6 mice^40^. Immediately after cell injection, the mice were randomly divided into two groups of seven each. Mice were then intragastrically (i.g.) administered with 0.5% Carboxymethyl cellulose-Na (CMC-Na) (vehicle control) or 1.2 g/kg (human equivalent dose) of SLE once per day for consecutive 15 days. To monitor the toxicity of SLE, general clinical observations were made once a day. Clinical signs, which included, but were not limited to, changes in skin, fur, eyes, mucous membranes, occurrence of secretions and excretions and autonomic activity (e.g., lacrimation, piloerection, pupil size, unusual respiratory pattern) were recorded. Changes in gait, posture and response to handling as well as the presence of colonic or tonic movements, stereotypes (e.g., excessive grooming, repetitive circling) or bizarre behavior (e.g., self-mutilation, walking backwards) were also recorded. Food and water consumption, and body weight of mice were measured once every 3 days. Tumor volumes were determined using a vernier caliper at days 7, 10 and 15 after cell injection. At the end of the experimental period, the tumor of each mouse was dissected and weighed. The organs including liver, kidneys, adrenals, testes, epididymides, spleen, brain and heart of each mouse were trimmed of any adherent tissue and weighed. Gross necropsy was performed for all the dissected organs and tissues. Proteins from tumor tissues were extracted with RIPA lysis buffer and examined by immunoblotting.

### Cell culture

Human A375 and murine B16F10 melanoma cells were obtained from American Type Culture Collection (ATCC, USA). Human keratinocyte cell line HaCaT and human adult dermal fibroblasts HDFa were obtained from Invitrogen (Casecade Biologics, Invitrogen cell culture, CA). A375, B16F10 and HaCaT cells were cultured in DMEM (GIBCO, USA) supplemented with 10% fetal bovine serum (FBS, GIBCO, USA) and 1% penicillin/streptomycin (P/S, GIBCO, USA). HDFa cells were cultured in medium 106 (GIBCO, USA) supplemented with LSSG kit (GIBCO, USA). All cells were maintained at 37 °C and in a humidified atmosphere of 5% CO_2_.

### Cell viability assay

MTT (3-[4,5-dimethylthiazol-2-yl]-2,5-diphenylterazolium bromide) assay^41^ was performed to determine the cytotoxicity of SLE on A375, HaCaT and HDFa cells. Cells (5,000 cell/well) seeded in 96-well plates were treated with SLE at various concentrations (0–1000 μg/mL) for 24 or 48 h. 20 μL of MTT solution (5 mg/mL) was added to each well and incubated for additional 4 h. The medium was then aspirated, and 100 μL of DMSO was added to dissolve the formed formazan crystals. The absorbance was measured at 570 nm by a microplate spectrophotometer (BD Biosciences, USA).

The cytotoxic effect of SLE on B16F10 cells was determined by trypan blue exclusion assay. Cells (1 × 10^5^ cell/well) were seeded overnight in 6-well plates, and subsequently treated with various concentrations of SLE (0–1000 μg/mL) for 24 or 48 h. After treatment, cells were collected, stained with 0.4% trypan blue, and counted using a Countess Automated Cell Counter (Invitrogen Japan K.K.). Crystal violet staining assay^41^ was also employed to visualize the effects of SLE on the proliferation of B16F10 cells. Treated cells were fixed with 10% formalin for 5 min, followed by staining with 0.05% crystal violet solution in distilled water for 30 min. Cells were then washed and photographed.

### Apoptosis assay

Apoptotic effects of SLE on A375 cells were quantified by Annexin V/7-AAD double staining assay^42^ with the Apoptosis Detection Kit (BD Biosciences, San Jose, CA, USA). After treated with SLE for 48 h, both detached and adherent cells were harvested and then incubated in 100 μL labeling solution (5 μL of AnnexinV-PE, 5 μL of 7-AAD, 10 μL of 10X binding buffer and 80 μL of H_2_O) in darkness for 15 min. 400 μL of 1X binding buffer was added to stop the staining reaction. Flow cytometric analysis was performed on a FACSCalibur^TM^ (BD, San Jose, CA).

### *In vitro* cell migration assay

The effect of SLE on cell migration was evaluated by wound healing assay^43^. Cells were allowed to grow to 80–90% confluence in 6-well plates. Cell monolayers were scratched with a sterile 10 μL pipette tip across the center of the well to generate a clean, straight wound area. Cells were then washed with PBS to remove the detached cells and incubated with vehicle (control) or SLE in serum-free DMEM medium. Cell migration was photographed at the 0 and 24 h time points with a digital camera installed on the microscope (Leica, Germany). Five images were taken of each well.

### *In vitro* cell invasion assay

Cell invasion was determined using BD BioCoat^TM^Matrigel^TM^ invasion chamber (24 well plate, 8 mm pore size) according to the manufacturer’s instructions^43, 44^. Invasion chambers were pre-hydrated for 1 h. 0.75 mL of DMEM with 10% FBS was added into the lower chamber. 1.5 × 10^5^ cells in 500 μL of DMEM-0.1% BSA with SLE or vehicle (control) were placed in the upper chamber. After incubation at 37 °C for 24 h, chambers were washed with PBS. The matrigel and cells remaining in the upper chamber were discarded by scrubbing with a cotton swab. Afterward, cells on the lower surface of the membrane were fixed with 4% paraformaldehyde and stained with crystal violet. Each chamber was washed 3 times with distilled water. Invaded cells (cells on the lower surface of the membrane) in five microscope areas (200 × magnification) were counted and imaged by a microscope (Leica, Germany).

### Western blot analysis

Whole cell lysates were prepared from cultured cells and melanoma tissues. Standard western blotting assay was performed as previously described^41^. Immunoreactive bands were visualized using the Enhanced chemiluminescence (ECL) detection system (Invitrogen, Carlsbad, CA, USA).

### Subcellular fractionation

Cells were washed once with cold PBS and scraped into 400 μL of hypotonic lysis buffer^23^. After incubation on ice for 15 min, 12 μL of Nonidet P-40 (NP-40) (10%, v/v) was added and cells were kept on ice for another 10 min. The supernatants were collected as the cytoplasmic extracts after centrifugation at 14,000 rpm at 4 °C for 1 min. The nuclei pellets were then washed once with hypotonic buffer and incubated on ice in high salt buffer for 30 min^23^. After centrifuging at 14,000 rpm at 4 °C for 10 min, the lysates were taken as nuclear fractions.

### Immunofluorescence assay

A375 cells were seeded on glass coverslips and incubated without or with SLE for 48 h. After treatment, cells were fixed with 4% formaldehyde in PBS for 15 min, permeabilized with ice-cold 100% methanol for 10 min and blocked in blocking buffer. Then the coverslips were incubated overnight with specific primary antibody against STAT3 (1:1600) at 4 °C. After washing with PBS, coverslips were incubated with fluorescein isothiocyanate (FITC)-conjugated secondary antibody (1:500) at room temperature in darkness for 1 h. Coverslips were then mounted on glass slides using Fluoroshield^™^ with 4′,6-Diamidino-2-Phenylindole (DAPI) (Sigma-Aldrich, USA). Images were viewed and photographed using a fluorescence microscopy (DMI 3000B, Leica, Wetzlar, Germany)^41^.

### Quantitative real-time polymerase chain reaction analysis (RT-PCR)

Total RNA was extracted with Trizol reagent (Invitrogen, USA) and reverse-transcribed into cDNA using PrimeScriptTM RT reagent Kit (Takara, Japan). Quantitative RT-PCR was performed in triplicate using iTaq™ Universal SYBR Green Supermix (Bio-Rad, USA) with a ViiA 7 Real Time PCR System (Applied Biosystems, USA). Quantification analysis was performed by the comparative C_T_ method^41, 45^. Relative gene expression was normalized to the endogenous control GAPDH.

### Stable transfection

A375 cells were transfected with pcDNA3-STAT3C plasmid or empty vector using Lipofectamine 2000 (Life Technologies, Inc., Rockville, MD) following the manufacturer’s instructions^40^. 72 h after transfection, cells were selected with 1 mg/mL of G418 in DMEM with 10% FBS for 14 days. G418-resistant colonies were pooled and regarded as STAT3C-expressing stable cells.

### Statistical Analysis

The data were expressed as mean ± SD and analyzed by one-way analysis of variance (ANOVA) followed by Fisher’s Least Significant Difference (LSD) Test using IBM SPSS Statistics Version 20.0 (IBM Corp., Armonk, New York). The difference was considered significant if *P* < 0.05.

## Electronic supplementary material

Supplementary Dataset
