# Supplementary figures and images for: Inhibiting STAT3 signaling is involved in the anti-melanoma effects of a herbal formula comprising Sophorae Flos and Lonicerae Japonicae Flos
Source: Sci Rep. 2017 Jun 8;7:3097. doi: 10.1038/s41598-017-03351-2 (PMC5465088; doi:10.1038/s41598-017-03351-2)

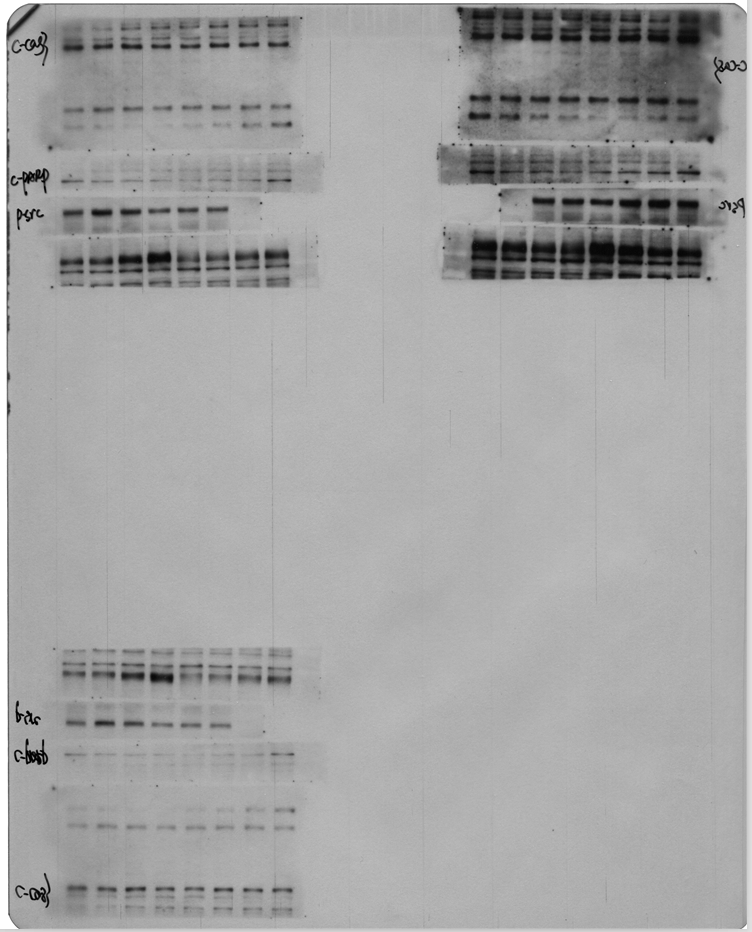

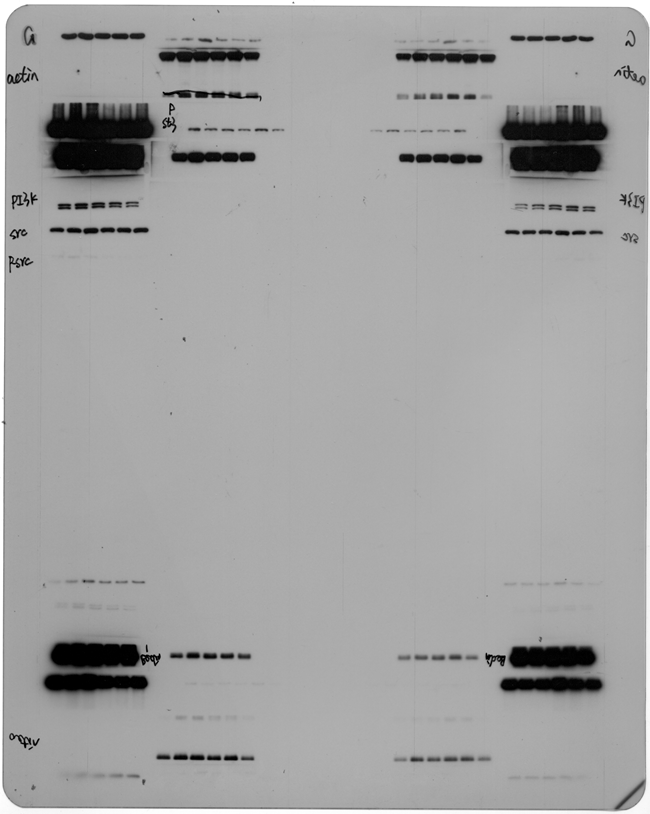

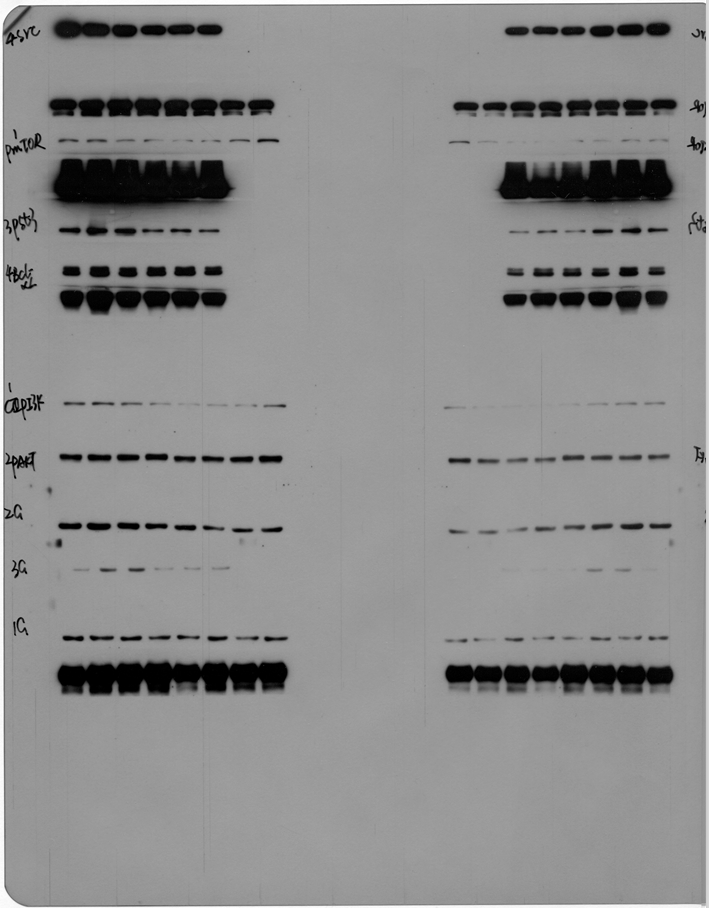

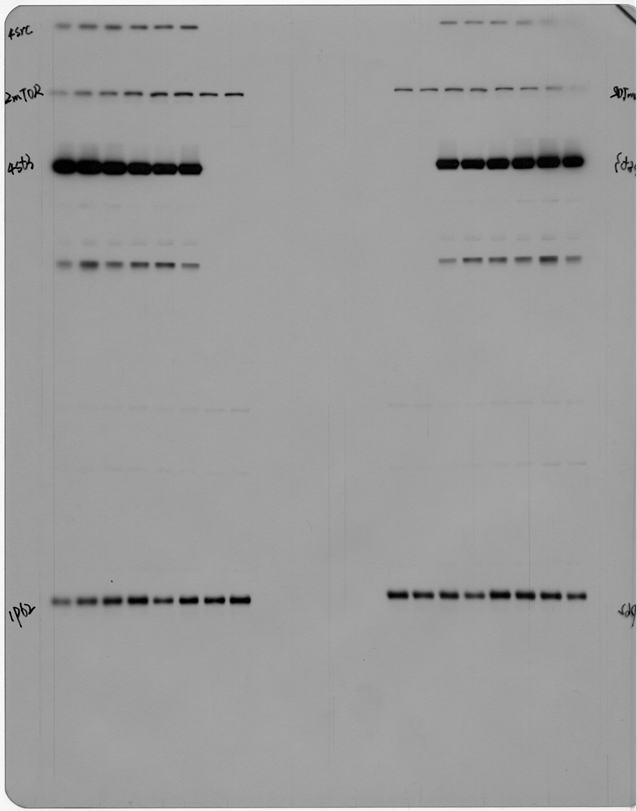

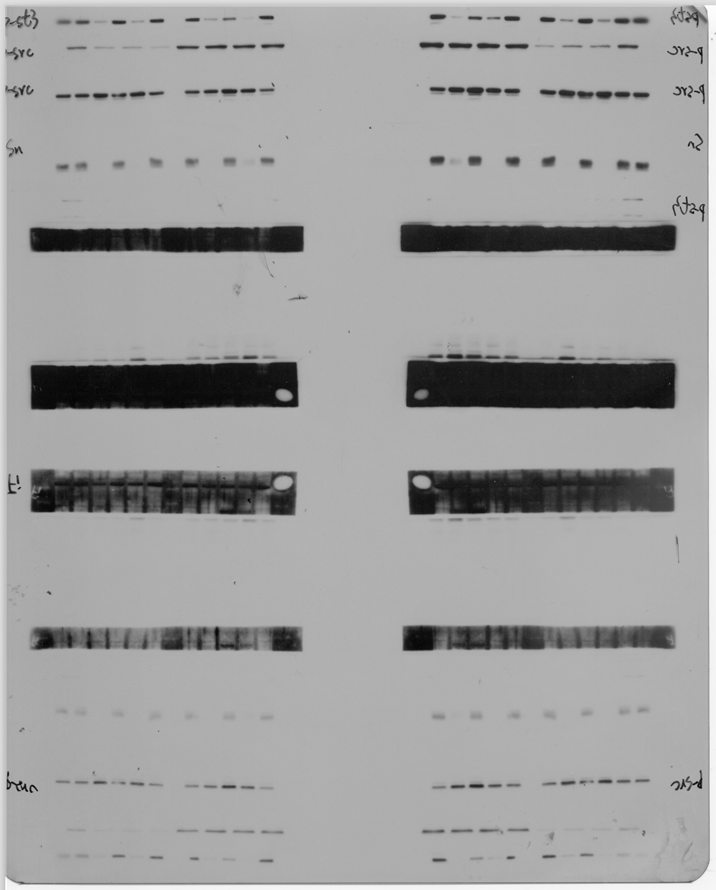

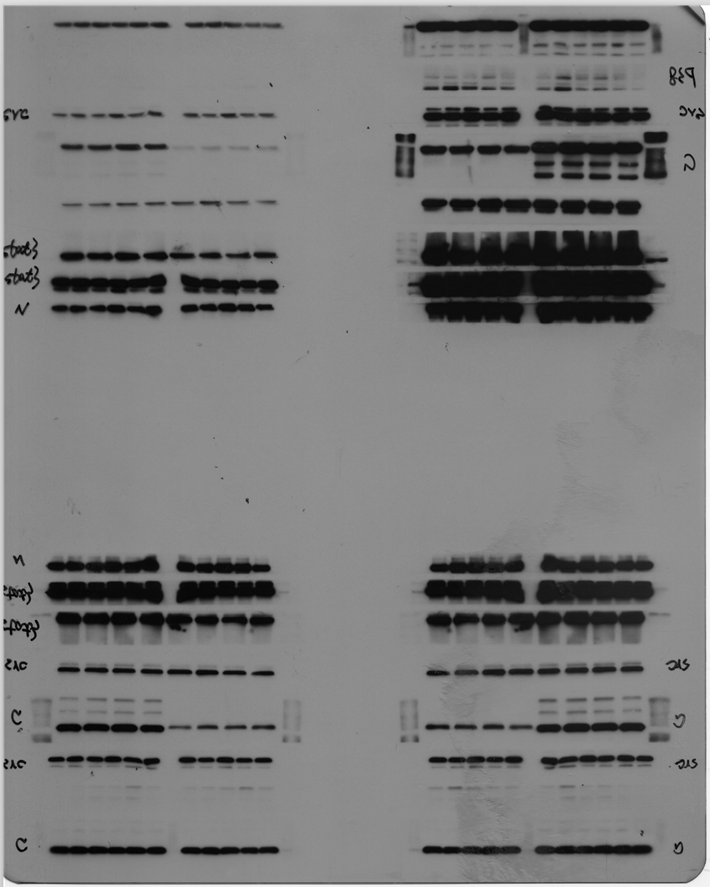

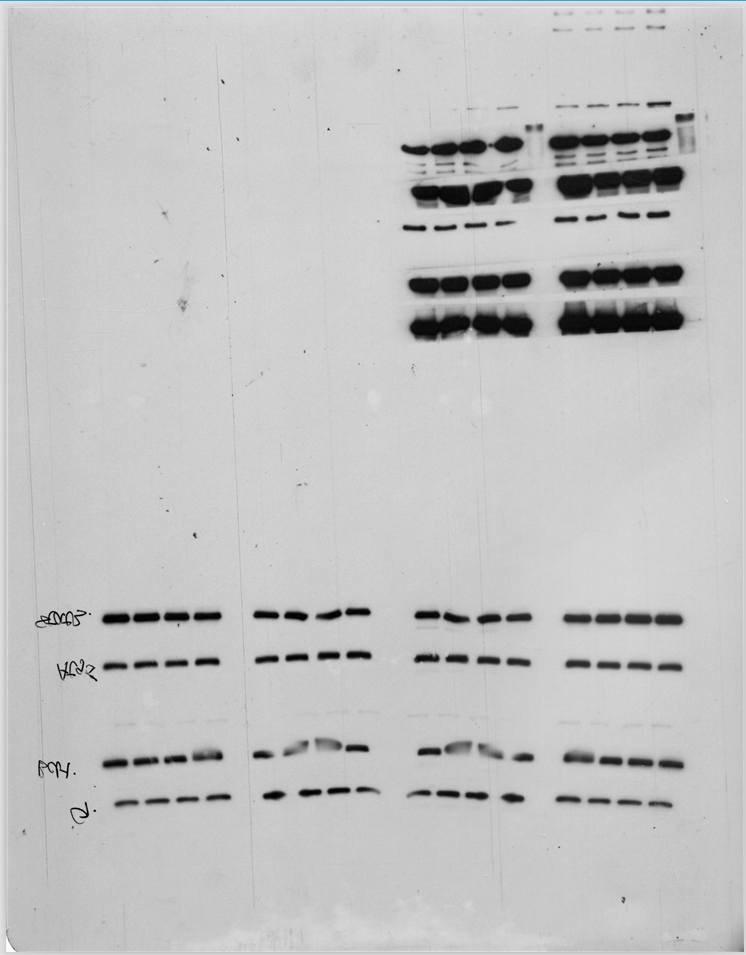

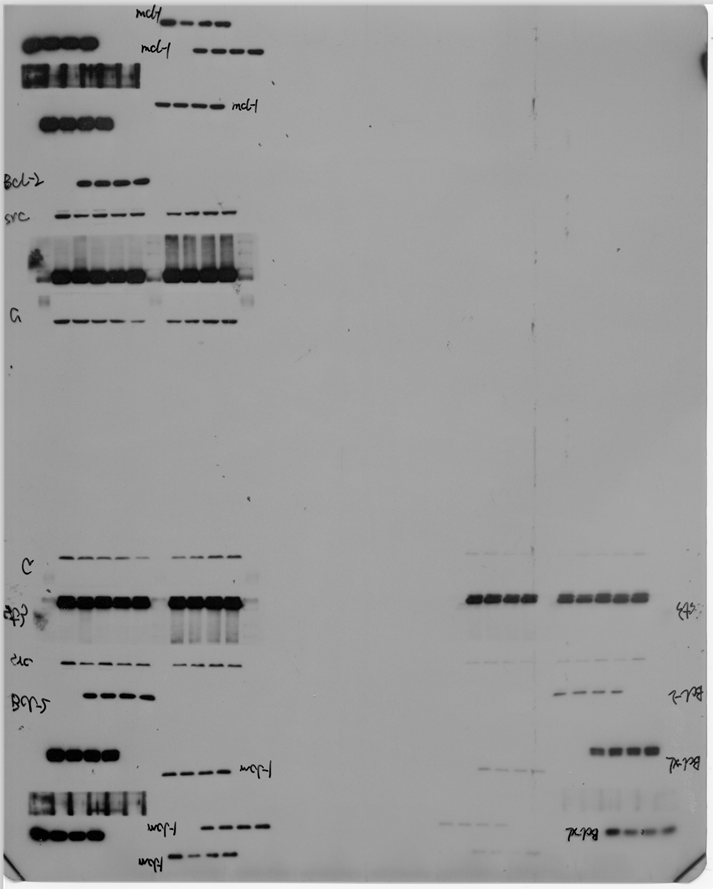

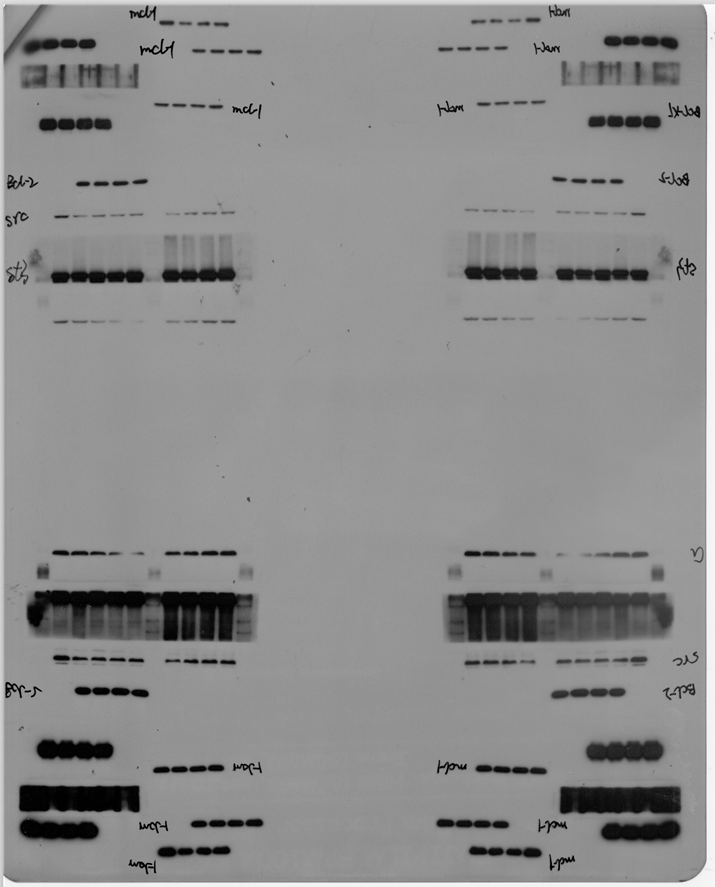

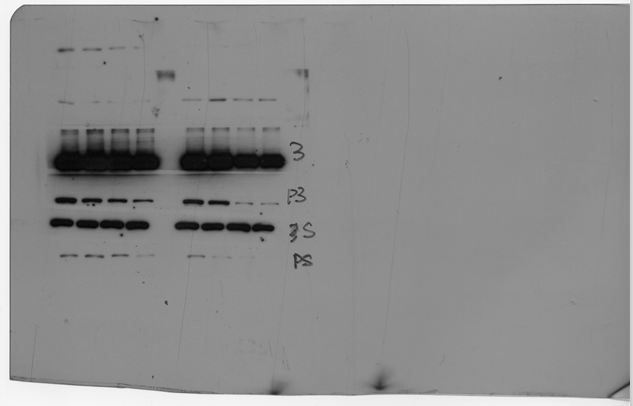

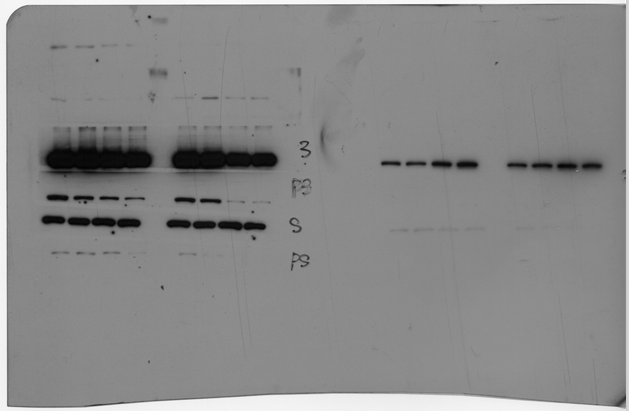

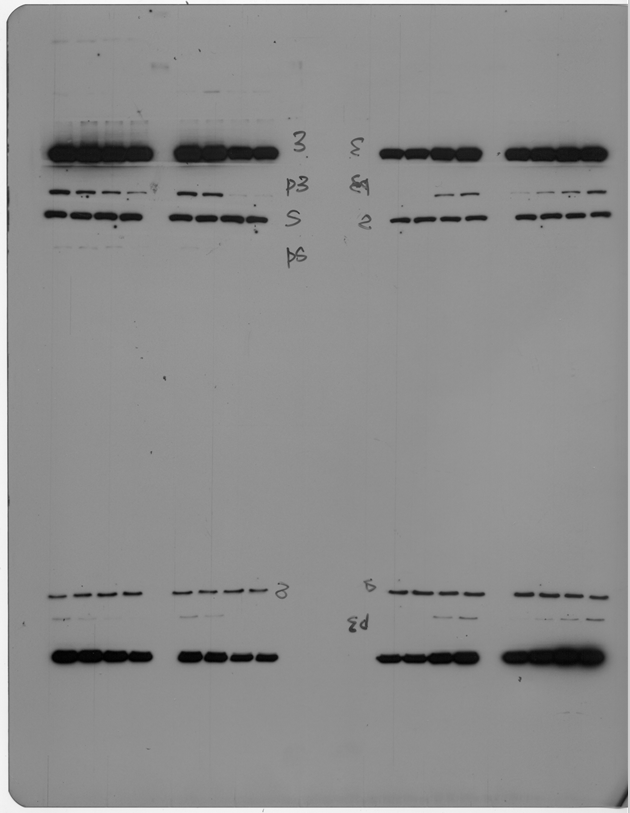

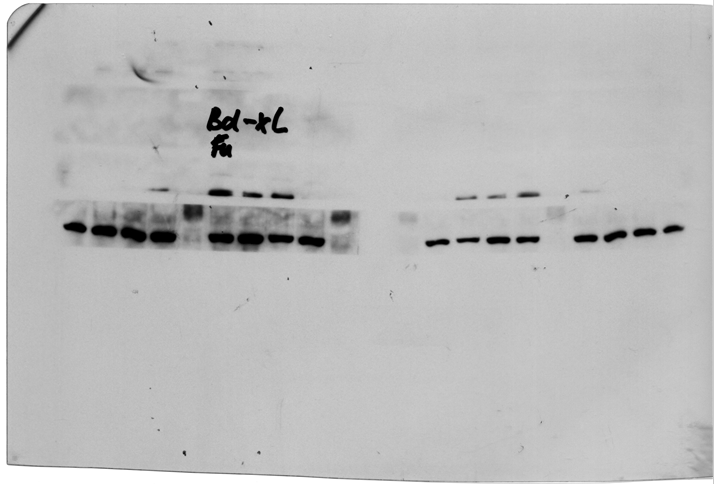

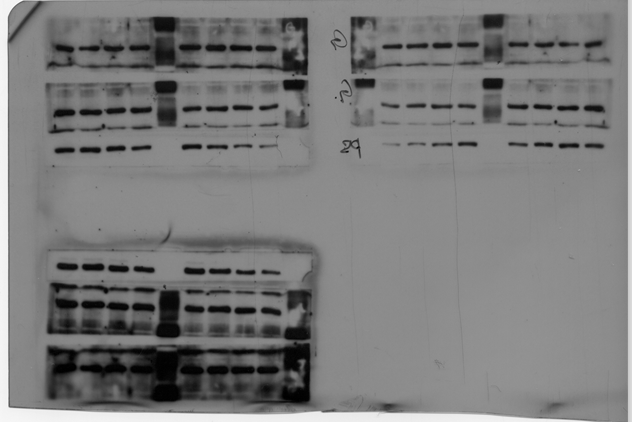

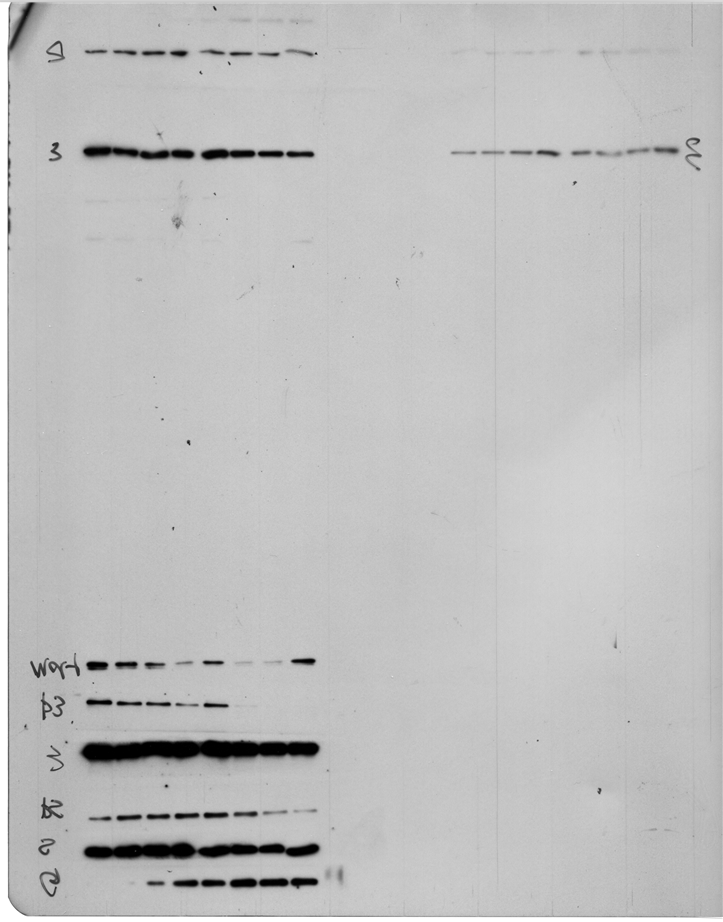

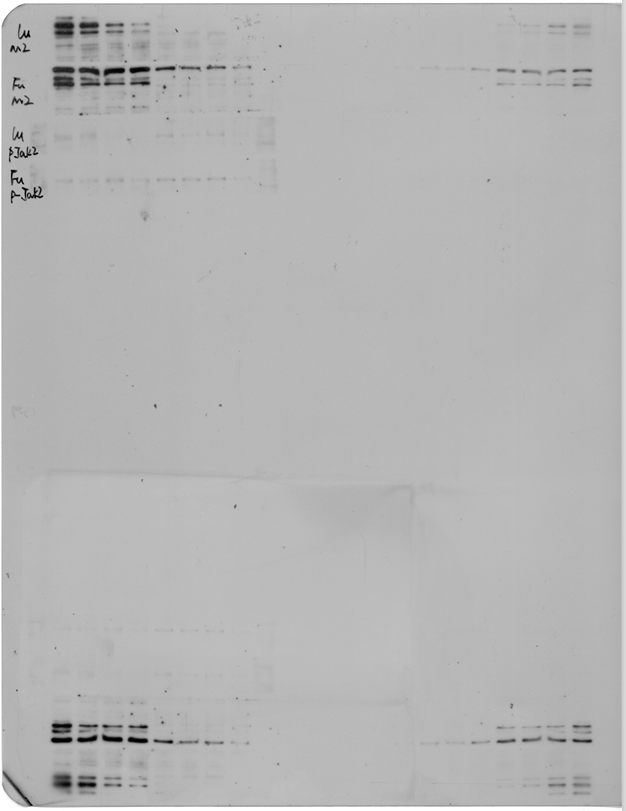

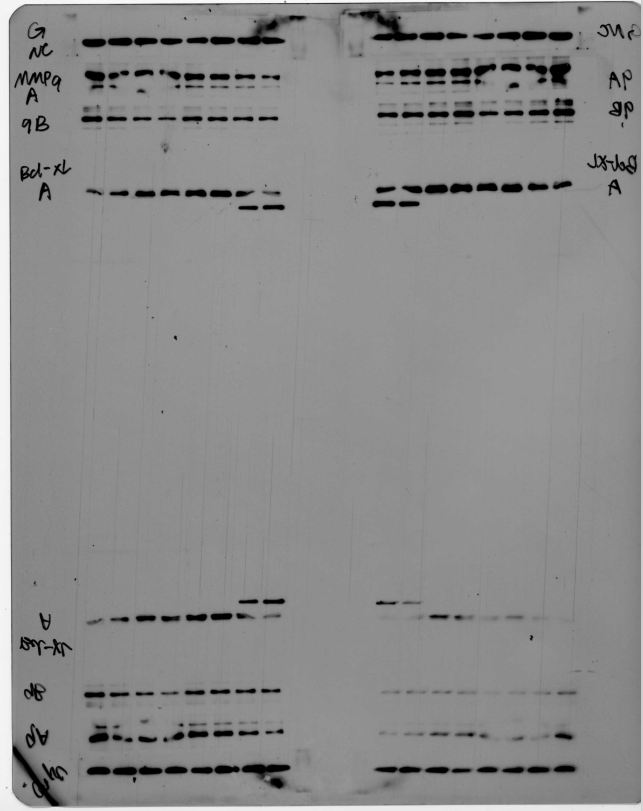

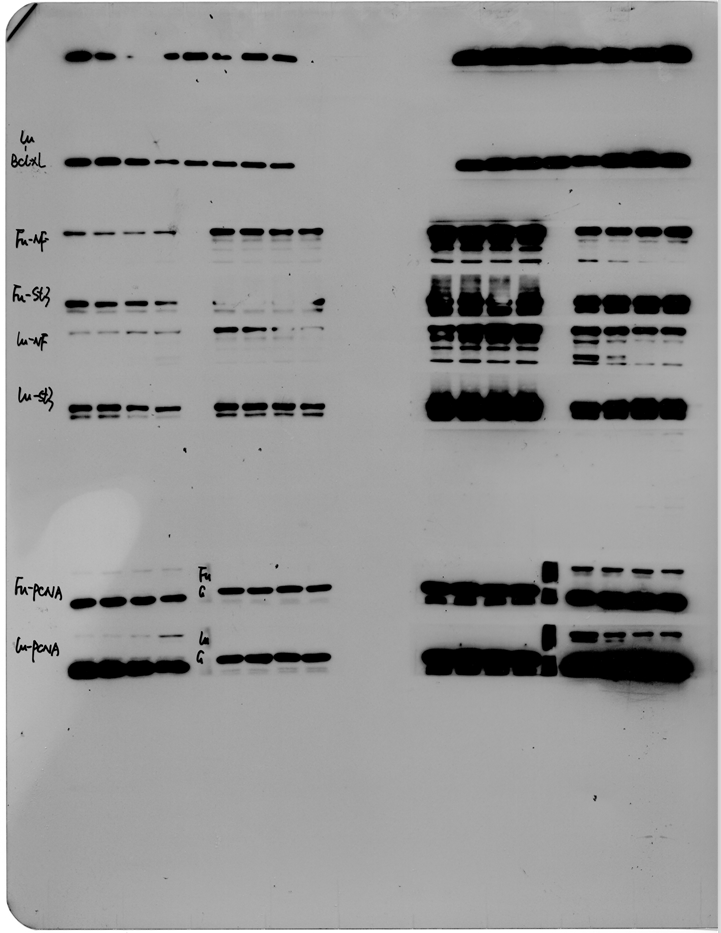

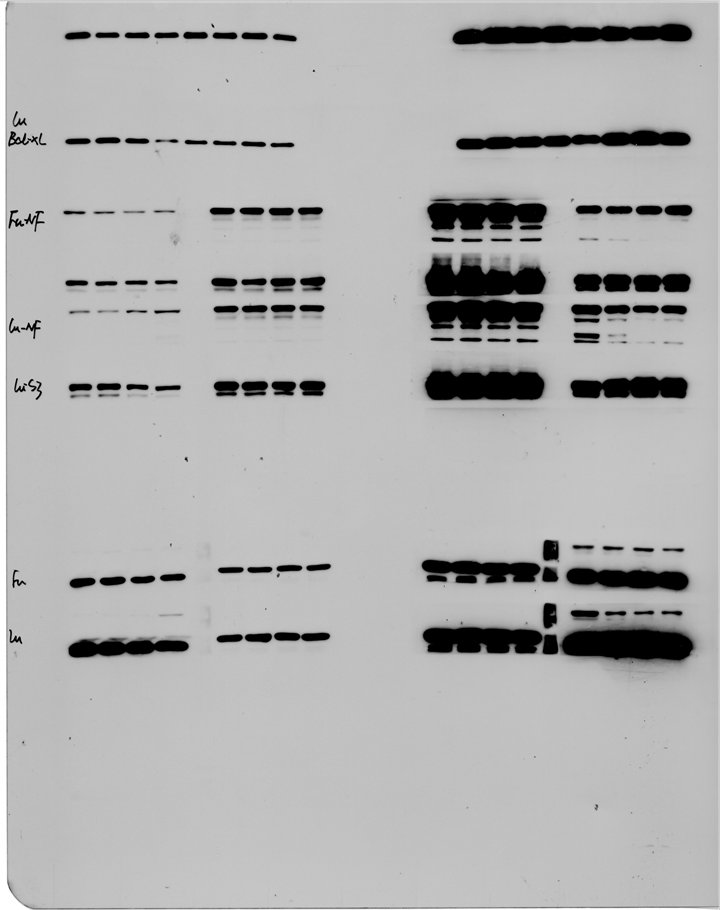

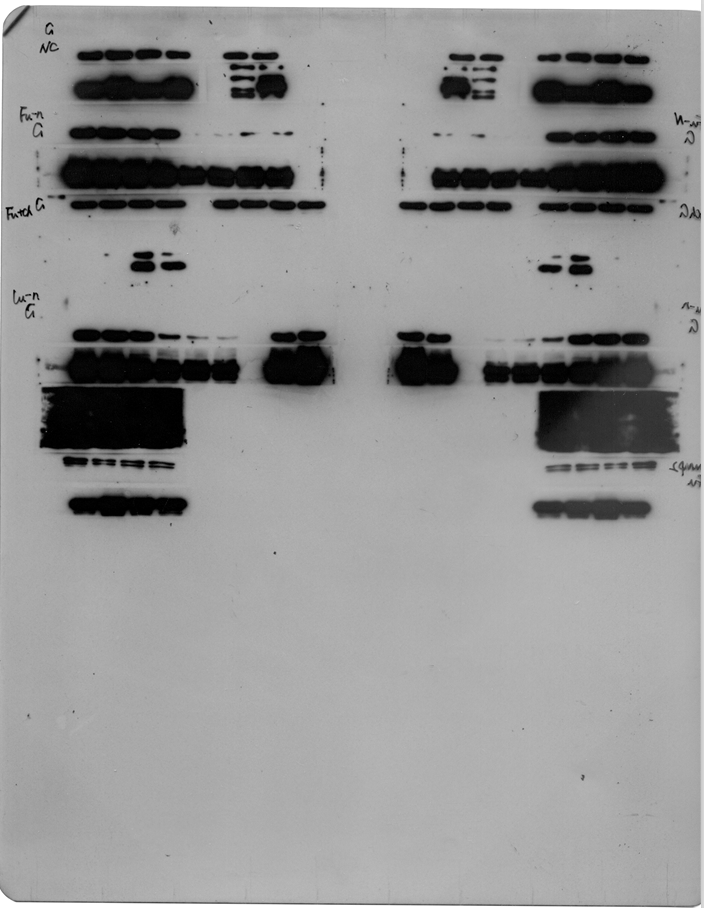

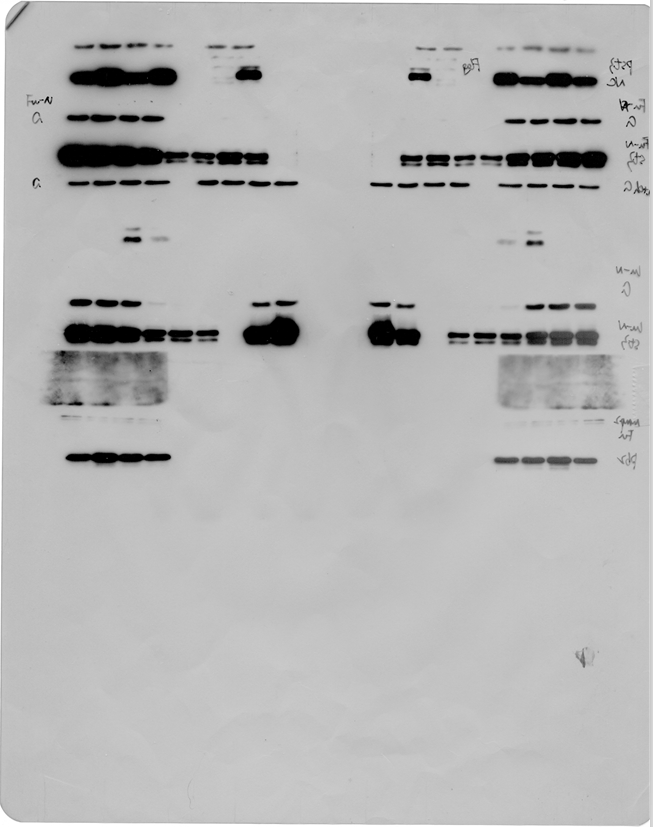

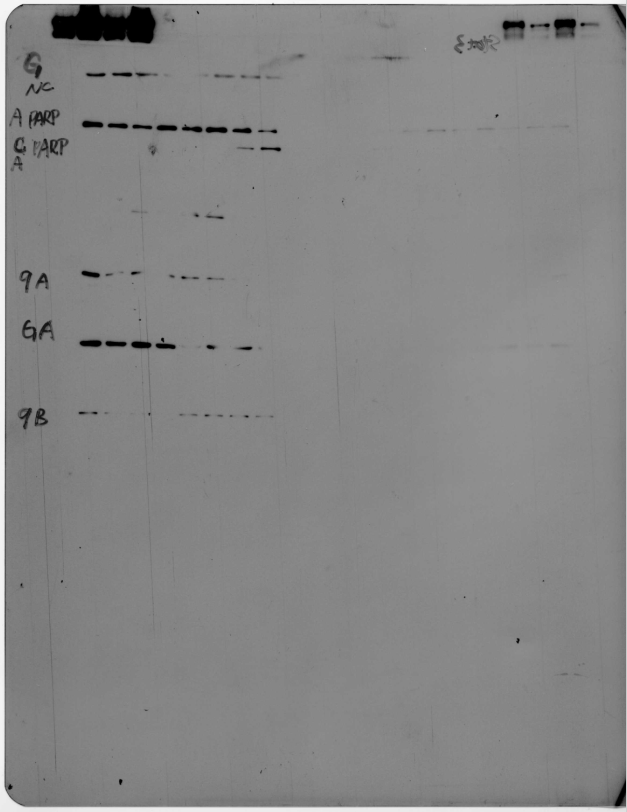

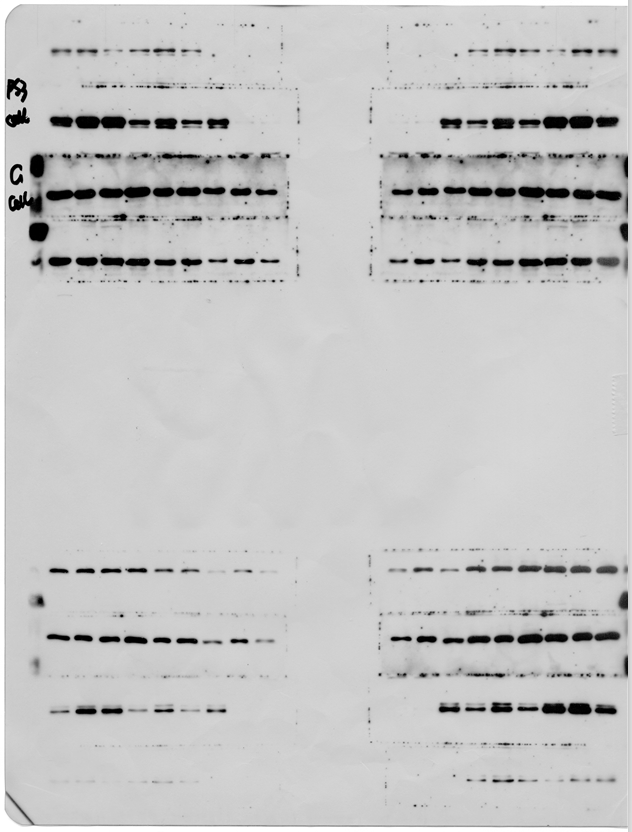

Supplement: Supplementary file 1 — Supplementary Dataset [file 41598_2017_3351_MOESM1_ESM.doc]
